# Leveraging pathway analysis in a human skin model of healthy aging

**DOI:** 10.18632/aging.204456

**Published:** 2022-12-24

**Authors:** Dimitrios Tsitsipatis, Myriam Gorospe, Allison B. Herman

**Affiliations:** 1 Laboratory of Genetics and Genomics, National Institute on Aging Intramural Research Program, National Institutes of Health, Baltimore, MD 21224, USA

**Keywords:** skin aging, GESTALT, network analysis, proteomics, primary skin fibroblasts

**Comment on:** Tsitsipatis D, et al. Proteomes of primary skin fibroblasts from healthy individuals reveal altered cell responses across the life span. Aging Cell. 2022; 21:e13609. PMID:35429111

Countless studies aiming to better understand human aging have been published in recent years. A surge of research seeks to improve the mean and maximum life span by identifying molecular and cellular events critical for the processes that drive aging. The skin reflects many of the physical and functional declines associated with aging, as evidenced by cutaneous thinning and sagging, loss of fat, and progressive inability to repair damage. Therefore, studying skin-derived cells offers a unique opportunity to identify global changes occurring with aging. Dermal fibroblasts, which typically produce skin collagens, fibronectins, elastin, and glycoproteins, decline both in number and biosynthetic capacity with age, in turn contributing to dermal atrophy and loss of ability to remodel the extracellular matrix (ECM) in older persons [1].

**Figure 1:**
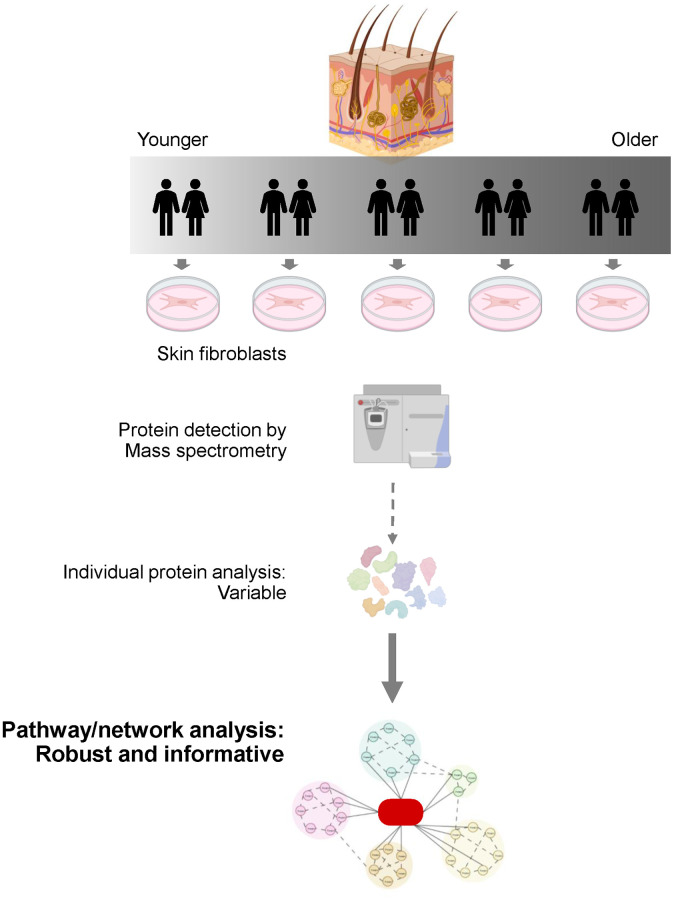
**Leveraging Pathway Analysis in a Human Skin Model of Healthy Aging.** Primary fibroblast cultures were established from skin biopsies of individuals within the GESTALT study of healthy aging (NIA, NIH), and their proteomes were analyzed by mass spectrometry. Although the expression levels of individual proteins varied across donors within the same age group, the activities of pathways in which these proteins participate were highly consistent. Hence, we propose that interventions aimed at restoring the function of pathways (instead of the function of individual proteins) may be more effective in preventing age-associated declines in older tissues.

We recently reported the changes in the proteome of primary skin fibroblasts derived from 82 ‘very’ healthy individuals (spanning 22–89 years old) who participated in the GESTALT (Genetic and Epigenetic Signatures of Translational Aging Laboratory Testing) study of the National Institute on Aging, NIH [2]. Following state-of-the-art mass spectrometry analysis, our study quantified 9,341 proteins, among which 268 proteins were significantly associated with aging. To shed light on the cellular processes changing across the healthy life span, we then focused on protein pathways significantly affected in older individuals. Among them, DNA replication and repair processes were most prominently declining with age, as illustrated by marked reductions in all six conserved members of the minichromosome maintenance (MCM) complex family (MCM2-7) in primary skin fibroblasts from older individuals [3]. Redox detoxification was also elevated, fostered by high levels of free-radical scavenging proteins like superoxide dismutase, glutaredoxins, and thioredoxins [4] in fibroblasts from older participants. Similarly, autophagy was significantly activated in this cohort; this finding was unexpected, as autophagy was often found to decline with advancing age [5], but elevations in autophagy were reflected in the proteomes of muscle biopsies from the same cohort [6], and in other evidence linking enhanced autophagy with healthy aging and longevity. These pathway predictions were validated by directly measuring readouts of these pathways—i.e., cell proliferation, DNA repair, reactive oxygen species accumulation, and autophagy—in a subset of individuals. These results revealed that analyzing pathway activity, instead of measuring the levels of individual proteins, is particularly informative when studying human aging (Figure 1), likely because there is redundancy in a key pathway, and the cell can implement or suppress them using different proteins.

Other interesting pathways were identified as being differentially represented among fibroblasts from different age groups, but await future experimental validation. For example, pathways related to Golgi organization and protein trafficking from the endoplasmic reticulum to the plasma membrane could explain the need to accommodate demands for increased secretion by senescent cells accumulating in the skin. Covalent histone modification, associated with the structural changes occurring during replication and transcription, were significantly reduced, and may explain the progressive impairment in DNA homeostasis and mRNA processing with age [7, 8]. Lastly, cell migration was also significantly underrepresented in older fibroblasts, potentially reflecting the hindered ability of dermal fibroblasts to assist in wound healing with advanced age.

Undoubtedly, across the body, cells in different tissues and organs will experience unique age-related changes; however, when given the opportunity to study aging directly from human samples, characterizing age-dependent pathways whenever possible, rather than individual proteins, appears to be far more informative. Importantly, interventions to restore the function of pathways hindered by aging, e.g., by increasing the activity of enzymes involved in DNA repair, restoring redox balance, or enhancing autophagy, offer relatively new and exciting means to prevent decline or rejuvenate tissues. Although cultured primary skin fibroblasts cannot faithfully recapitulate certain aspects of human skin, such as oxygen tension, substrate stiffness, and different neighboring cell types, they can serve as a simplified model to study certain aspects of human skin aging, investigate pathways differentially active as a function of age, and test prominent interventions before evaluating their effectiveness for promoting healthy aging *in vivo*.
